# How adolescent and young adult cancer is portrayed in movies: reflections for supportive care

**DOI:** 10.1007/s00520-026-11138-3

**Published:** 2026-08-31

**Authors:** Meredith K. Reffner Collins, Rachel S. Anca, Brinda Pusuloori, Dawn Carey, Karen Long-Traynor, Archana Sharma, Katie A. Devine

**Affiliations:** 1 Department of Pediatric Hematology/Oncology, Rutgers Cancer Institute, 195 Little Albany Street, New Brunswick, NJ 08903 USA; 2 Johns Hopkins Bloomberg School of Public Health, Baltimore, MD USA

**Keywords:** Survivorship, Adolescent and young adult cancer, Psychological well-being, Psychosocial support systems, Social communication, Health communication

## Abstract

**Purpose:**

Adolescent and young adult cancer survivors (AYACS) often experience psychological distress caused by difficulty in maintaining relationships. Cancer-related movies could promote knowledge and self-efficacy in relationship maintenance, but little is known about how cancer is depicted on screen. This study explored how the medical and social consequences of cancer are depicted in movies featuring an AYA with cancer.

**Methods:**

We conducted a content analysis of cancer-related movies released between 2013 and 2023. Two independent coders collected information about each character with cancer, each medical scene, and each social scene.

**Results:**

The final sample included 20 movies and 24 AYA characters with cancer. These characters were primarily female, White, and heterosexual, and more than half died during the movie. Only about 30% of medical scenes showed characters receiving any kind of cancer treatment. The most prevalent social theme was related to death. The receipt and provision of informal, non-medical care was the second most frequent social theme. Survivorship themes accounted for less than 10% of the sample.

**Conclusions:**

Popular movies featuring depictions of AYA cancer are mostly not reflective of the cancer experience, especially as related to treatment and survival. The inclusion of themes related to the receipt and provision of informal care was noteworthy for its alignment with the existing literature. Movies may provide an opportunity for survivors to start conversations about preferences for the receipt and provision of non-medical support.

**Supplementary Information:**

The online version contains supplementary material available at https://doi.org/10.1007/s00520-026-11138-3.

## Introduction

Approximately one in three adolescent and young adult cancer survivors (AYACS; ages 15–39 years) experience psychological distress, including elevated levels of depression and anxiety, even after treatment is completed [1, 2]. AYACS risk for psychological distress, compared to their older and younger counterparts, is greater due to the unique challenge of facing a cancer diagnosis alongside the developmental tasks that occur during this life stage [1, 3]. Managing the late and long-term physical and psychosocial consequences from cancer extends the risk for distress well past the end of treatment [4].

Unwanted changes in peer relationships are an important component of AYACS psychosocial distress, as cancer can interrupt and negatively affect these key relationships [5, 6]. Approximately two-thirds of AYACS report experiencing either negative support, such as trivialization or invalidation of their experiences, or a lack of support, such as withdrawal from the relationship [7–11]. AYACS often report fear of feeling unheard or misunderstood, and they adjust their behavior accordingly by limiting or withholding cancer-related feelings [9, 12–14]. These changes in peer relationships can cause loneliness and isolation, both of which are positively associated with increased levels of stress, depression, and anxiety [7].

Relationships involve more than one person, and healthy peers face their own set of difficulties. Friends of AYACS frequently describe avoiding support opportunities because they feel uncomfortable and lack confidence in their ability to help [8, 15, 16]. This perceived lack of self-efficacy is likely due to age, as healthy friends of AYACS often have limited or no experience with cancer and the healthcare system. Theoretically, if friends perceive the support they have provided to be ineffective, their willingness to offer future support could diminish, which could compound existing issues within the relationship [16–18].

To mitigate negative changes in AYACS and peers’ relationships — and AYACS downstream psychological distress — it is crucial to promote both parties’ knowledge, self-efficacy, and comfort in engaging in relationship-sustaining activities, such as holding cancer-related conversations and spending time together [12–14, 17]. Entertainment media narratives, including movies, books, and television series, could help facilitate these activities. Prior research has demonstrated that AYACS start cancer-related conversations with friends through entertainment media narratives [19–21]. Furthermore, shared consumption of entertainment media is already a part of the social context of this developmental stage. Adolescents and young adults report engaging with friends before, during, and after consuming media; they also describe using entertainment media as inspiration or idea generation when facing socialization issues [22–25]. Considering this context, it is possible that cancer-related movies could be useful in facilitating cancer-related conversations between AYACS and their friends. In fact, these depictions of cancer in entertainment media are likely the only exposure to cancer that both AYACS and their friends have prior to a diagnosis, thus making them a natural entry point into a conversational topic that is rare and uncomfortable for most young people [19, 26].

Co-viewing, or watching the same movie at the same time, can prompt conversation through several mechanisms. Co-viewing creates a shared base of material from which viewers can relate and integrate into their own lives; it also prompts opinion-sharing about content [24, 25]. Engaging in these types of interactions can thus serve as a natural catalyst for conversations between AYACS and their peers. Co-viewing a cancer movie may also eliminate the perceived hurdle of initiating a conversation about cancer. However, little is known about what type of content is in movies about cancer, especially those depicting instances of AYA cancer. Previous studies of pediatric and adult cancer-related movies have demonstrated that on-screen characters with cancer are often presented with terminal prognoses, and many die during the movie [27, 28]. Popular movies also often fail to showcase the multidisciplinary nature of modern cancer care, including the exclusion of critical support services such as psychosocial and palliative care [27, 28]. While the goal of these movies is for entertainment rather than complete accuracy, understanding what types of cancer-related depictions AYACS and their friends might encounter in movies can assist in the development of adequate psychosocial education and support.

The present study addresses this knowledge gap by conducting a content analysis of the medical and social content in AYA cancer-related movies. Because cancer is first and foremost a disease, analyzing how the medical aspects of the cancer diagnosis, treatment, and survivorship experience are depicted could be key to the development of future education and support resources. Additionally, because AYACS report significant impact of cancer on their psychosocial health — and because relationships are inherently social — analyzing how the social aspects of the cancer diagnosis, treatment, and survivorship experience are depicted could also be important for the development of future education and support resources [5–15]. Though the literature suggests multiple relevant theoretical frameworks, there was not one relevant unifying one. Thus, because our goal with this study was primarily descriptive and exploratory, we felt that adopting a literature-driven approach to this study was the most appropriate and we did not adopt a specific theoretical framework for the study. Our study, then, focused on the following two Research Questions:

RQ1: What are the features of medical scenes depicted in cancer-related AYACS movies?

RQ2: What are the features of social scenes depicted in cancer-related AYACS movies?

## Methods

### Sample

To identify a sample of AYACS movies, the inclusion criteria were (1) cancer must be the primary storyline in the film, regardless of genre; (2) the primary character with cancer in the movie was diagnosed with cancer between the ages of 15 and 39 years old and was still between 15 and 39 years (i.e., the AYACS age range [29]; if unspecified in the movie, the age of the actor or actress portraying the character was used); (3) movie release date was between January 1, 2013 and December 31, 2023; and (4) movie was set in the USA due to our interest in the portrayal of the healthcare system in the USA.

### Procedure

#### Codebook development

To code the movies that would comprise our sample, we developed a codebook a priori. Aligned with study objectives, we devised a procedure focused on medical and social scenes, and we based our initial codebook on the existing literature describing medical and social issues experienced by AYACS.

We defined a *medical scene* as one in which a character is shown interacting with medical personnel or a medical setting, receiving cancer-directed therapies and/or psychosocial support, and/or experiencing symptoms related to the presence of cancer or cancer treatment.

We defined a *social scene* as one in which a character or characters face a challenge, caused by the presence of cancer, in interacting with other characters or social institutions. To code these scenes thematically, two coders first wrote an open-ended written description of each social scene. These descriptions noted the social challenge faced, the characters involved in the challenge, and the effects of the challenge on the relevant characters. To assist with the identification of social scenes, a list of representative social challenges was developed based on the existing literature, including examples such as cancer “ghosting,” the use of dark humor, planning for eventual death and saying goodbye to others, receiving unsolicited medical advice, and parents taking care of a previously self-sufficient adult [7, 10, 11, 18, 30]. Disagreements about the extracted descriptions were resolved using a consensus-building approach. Then, two coders applied one or more of the 24 unique social codes to the extracted descriptions.

Prior to coding the sample, the coders (MKRC, BP, and RSA) familiarized themselves with all parts of the codebook using four practice movies not included in the final sample. Discrepancies were resolved using a consensus-building approach throughout all parts of the codebook, and minor adjustments were made.

#### Coding process

Because this study did not involve human subjects, no ethical approval was required. Access to each of the 20 films was secured through commercially available methods (streaming services, libraries, etc.).

Coding the final sample occurred in two phases. In the first phase, two trained coders (MKRC and BP) independently coded each of the 20 movies in a randomly generated order. Coders applied the quantitative codes covering the characteristics of the character with cancer and the medical scenes. Then, the two coders (MKRC and BP) created written descriptions of each social scene. The coders met once each week to review each movie and resolve discrepancies in the cancer characteristics and medical codes applied and in the extracted social scene descriptions. Discrepancies between coders (MKRC and BP) at this stage were resolved using a consensus-building approach; intercoder reliability for cancer characteristics, medical scene characteristics, and descriptions of social scenes was not calculated. Once all 20 movies had been coded in the first phase, two coders (MKRC and RSA) independently applied the thematic codes to the extracted social scene descriptions. Because a new coder (RSA) joined the team at this stage of the project, we decided to take the extra step of calculating interrater reliability using Gwet’s AC_1_ for these thematic social scenes [31–33]. Exactly half of the movies in the sample (*n* = 10, 50.0%) were double-coded, representing 58.0% of the total number of unique social scenes within the sample (*n* = 217 vs. *N *= 374). Agreement values for all but three coding categories were ≥ 0.90; in the remaining three categories, agreement values were still in the acceptable range at 0.86, 0.87, and 0.89 (see Table 4) [32]. We resolved any disagreements for final codes using a consensus-building approach. Due to these acceptable agreement values, we single-coded social scenes from the remaining 10 movies, with each coder coding five movies.

### Codes

Our codes categorized information across four domains for each movie: (1) Movie facts, (2) Cancer characteristics, (3) Medical scenes, and (4) Social scenes. Exact codes can be found in the codebook in Supplement 1. Importantly, medical scenes and social scenes were not necessarily independent of one another; there could be social challenges depicted within a larger medical scene (e.g., a character accompanying the character with cancer to treatment), and there could be medical components within a social scene (e.g., a character needing to stop during a diagnosis disclosure to vomit). For scenes that overlapped, the start time was defined as when the social or medical component was introduced, and the end time was defined as when the social or medical component was no longer present. Multiple codes could be applied per scene; however, each code represented a distinct action or thought within each scene.

#### Movie facts

Facts about each movie were recorded from the IMDBPro database and were current as of February 2024. Categories included the release year, genre classification, rating, movie length, director, and production and distribution companies. The IMDB MOVIEmeter ranking was also collected from the database. The MOVIEmeter ranking is an IMDBPro proprietary movie-ranking algorithm, and it calculates the popularity of any given movie based on the page views of a sampling group of more than 200 million monthly visitors [34]. A lower number indicates greater popularity.

#### Cancer characteristics

We also coded information about the character(s) with cancer and their diagnoses in each movie. We focused on demographic characteristics, such as the race, gender, and sexual orientation of each affected character. We noted the diagnosis, prognosis upon the introduction of the cancer diagnosis, and the characters’ vital status at the end of the movie. Diagnoses were collected exactly as they were given within the movie; movies that used “cancer” without additional information were coded as “cancer not specified.” Prognoses were also collected exactly as they were given in the movie; “ambiguous” was used when the prognosis was not clearly stated. Importantly, prognosis was coded at the time that the cancer diagnosis was introduced within the movie, and, in many of the films, a prognosis was not given along with a diagnosis. Finally, we coded the vital status of the character(s) with cancer at the end of the movie.

#### Medical scenes

In addition to noting the start and end time of each scene, we coded the setting(s), treatment type(s), and symptom(s) shown in each scene (see Supplement 1).

#### Social scenes

In addition to noting the start and end time of each scene, coders coded the descriptions of each social scene with one or more of 24 unique codes that could be collapsed into six overarching categories, including *physical concerns*, *processing*, *survivorship*, *death salience*, *caring*, and *interpersonal interactions* (see Supplement 1).

## Results

**Fig. 1:**
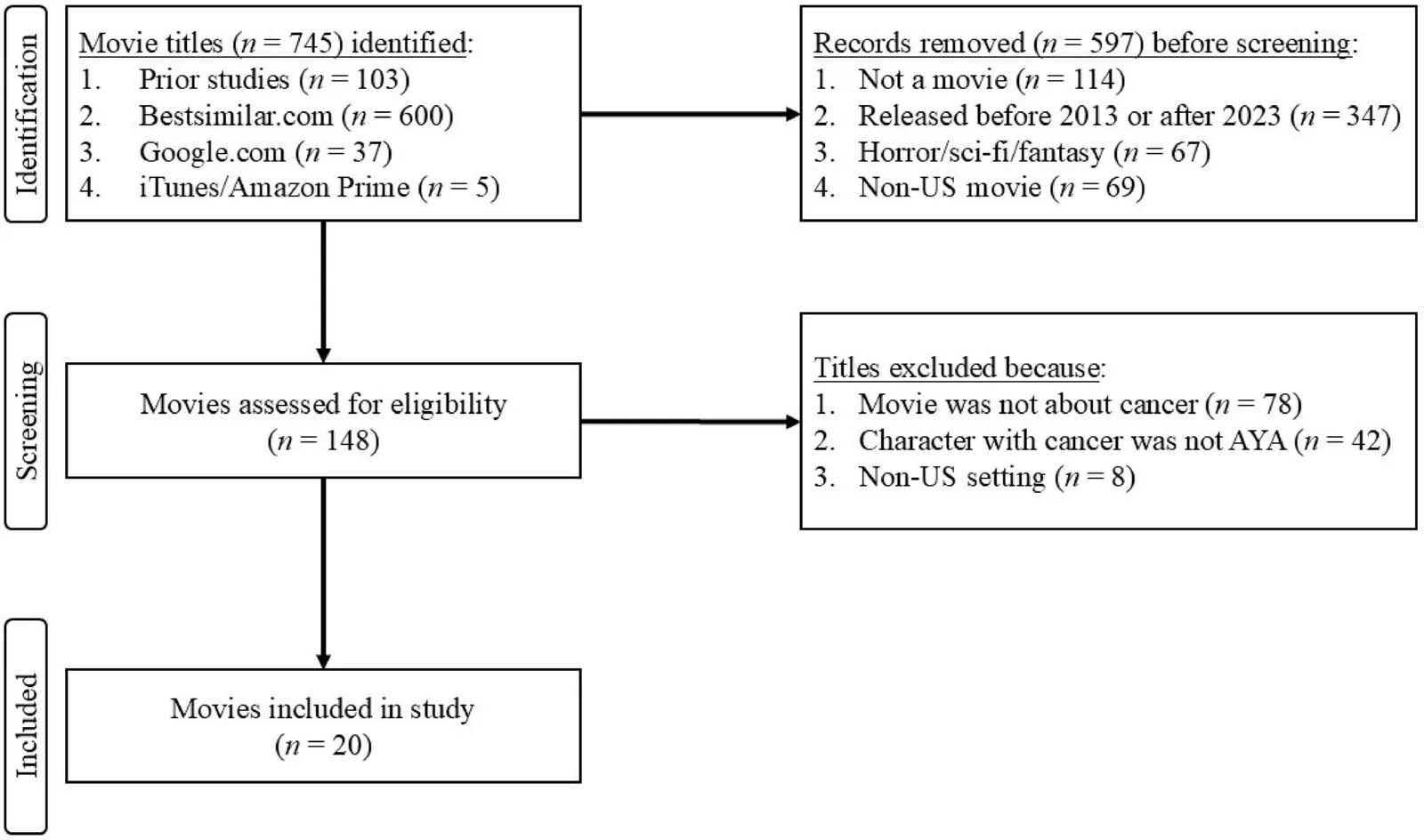
Movie title selection process

In total, we identified more than 700 potentially relevant titles (Fig. 1). To identify relevant titles, we first consulted prior studies [19–21]. Then, in February 2024, we used the “cancer” tag on the movie website BestSimilar [35] and the search string “adolescent and young adult cancer movies” on Google. After removing 597 ineligible titles (based on release date, genre, character age, and setting), the remaining 143 potentially eligible titles were fed into iTunes and Amazon Prime for “similar” recommendations, which netted five unique, additional titles. Throughout this process, we signed out of our personal Google, iTunes, and Amazon Prime accounts, we reset our Internet activity after every session, and we used private web browsers (i.e., “Incognito Mode”) to account for the potential influence of account personalization on the recommendations. The first author screened the remaining 148 movie titles by reading synopses of the movies’ plot(s), character(s), and setting(s) using the movie-specific Wikipedia article and the information about each title contained in the IMDBPro database [34].

The final sample included 20 movies (see Table 1). Run times of the movies ranged from 78 to 124 min (*M* = 101.1, *SD* = 13.6). There were 24 unique AYACS in the sample; two additional characters with cancer appeared in the movies, but they were not within the AYA age range and thus not included in the final sample. The 24 AYA characters with cancer (Table 2) were primarily female (*n* = 15, 62.5%), White (*n* = 19, 79.2%), and heterosexual (*n* = 22, 91.2%). About a third of characters suffered from an unspecified cancer (*n* = 8, 32.0%), and most prognoses were ambiguous (*n* = 13, 54.2%). Most characters with cancer were dead by the end of the movie (*n* = 14, 58.3%). The remaining characters were still alive by the end of the movie (*n* = 10, 41.7%), although only a small handful had explicitly reached no evidence of disease (*n* = 5, 20.8%).

**Table 1:** Movies included in sample (*N* = 20)

Movie title	Year released	Rating	Length (mins.)	IMDB Pro Movie Meter^a^	Medical scenes	Medical scenes total time (mins.)	Social scenes	Social scenes total time (mins.)
					*n*		*n*	
After Everything	2018	Not rated	95	13,959	16	17.5	22	46.7
After the Rain	2016	PG-13	95	98,093	13	23.6	20	42.9
All My Life	2020	PG-13	91	10,470	8	11.8	14	41.6
Clouds	2020	PG-13	121	15,780	11	16.0	27	73.4
Her Only Choice	2018	PG	90	175,961	15	36.6	15	34.9
Honeyglue	2015	R	107	110,537	15	18.8	14	40.2
Hooking Up	2020	R	104	24,795	4	2.3	20	51.4
Hope Springs Eternal	2018	PG	78	130,601	8	14.5	23	48.7
I Still Believe	2020	PG	116	10,857	11	26.1	15	49.7
Irreplaceable You	2018	TV-MA	96	19,692	16	21.3	25	68.6
Life in a Year	2020	PG-13	107	11,698	7	21.9	19	52.6
Me and Earl and the Dying Girl	2015	PG-13	105	7596	4	15.3	19	67.5
Meet My Valentine	2015	PG	85	88,346	9	12.7	16	61.7
My First Miracle	2015	PG	111	105,885	9	46.5	13	50.8
New Life	2016	PG	88	68,377	6	13.8	12	30.8
Our Friend	2019	R	124	6995	12	21.8	21	61.6
Sequoia	2014	Not rated	86	94,948	4	22.7	17	50.7
The Fault in Our Stars	2014	PG-13	126	2745	15	126.0	22	96.2
Then Came You	2018	Not rated	97	17,942	6	17.2	19	56.3
Until Forever	2016	Not rated	100	49,405	12	33.4	21	76.2
*M* (*SD*)			101.1 (13.6)	53,234 (51,594)	10 (4)	26.0 (25.4)	19 (4)	55.1 (15.6)

^a^As of February 15, 2024, lower numbers signal greater popularity. On the same day, these recently released and/or award-nominated movies were in the Top 10: # 2: *Argylle*, 2024; # 4: *Poor Things*, 2024; # 7: *The Beekeeper*, 2024; # 8: *Saltburn*, 2023

**Table 2:** Adolescent and young adult (AYA) characters in the sample (*N* = 24)

	Number	Percent
Type of character		
Leading	15	62.5
Supporting	9	37.5
Race		
White	19	79.2
Black or African-American	4	16.7
Asian	1	4.2
Gender		
Female	15	62.5
Male	9	37.5
Sexual orientation		
Heterosexual	22	91.7
Not heterosexual	1	4.2
Unclear	1	4.2
Diagnosis		
Not specified	8	33.3
Bone/soft tissue	5	20.8
Blood	3	12.5
Breast or testicular	3	12.5
Tumor	3	12.5
Brain/eye	2	8.3
Prognosis		
Ambiguous	13	54.2
Terminal	9	37.5
Curable	1	4.2
Odds given	1	4.2
Final state of character		
Dead	14	58.3
Remission/no evidence of disease	5	20.8
No change	4	16.7
Unclear	1	4.2

Two additional characters with cancer were not within the AYA age range

### Medical scenes

There were 201 unique medical scenes within the sample. Each movie averaged 10 medical scenes, and screen time devoted to medical scenes ranged from 2.3 to 126 min (*M* = 26.0, *SD* = 25.4, median = 20.0). Within these scenes, there were 108 symptoms and 62 treatments shown (Table 3).

**Table 3:** Symptoms and treatments depicted in medical scenes (*N* = 201)

Symptoms	Number	Percent
Number of symptoms per medical scene (*N *= 201)		
No symptoms present in scene	93	46.0
1	59	29.4
2	31	15.4
3 or more	18	9.0
Symptom type (*N *= 108)		
Pain or trouble moving around	36	33.3
Trouble breathing or reduced function of other systems (cardio, pulmonary, speech, etc.)	30	27.8
Other^a^	27	25.0
Changes to skin or nails	23	21.3
Fatigue or irritability/mood swings^b^	19	17.6
Nausea/vomiting/other gut issues	16	14.8
Bleeding, bruising, swelling, or lumps	9	8.3
Hallucinations or problems with vision or hearing	9	8.3
Coughing	8	7.4
Dizziness or fainting	8	7.4
Hair loss	7	6.5
Treatments	*n*	%
Number of treatments per medical scene (*N *= 201)		
No treatments present in scene	139	69.2
1	55	27.4
2 or more	7	3.5
Treatment type (*N *= 62)		
Chemotherapy	27	43.5
Pills^c^	15	24.2
Support group	9	14.5
Other	8	12.9
Surgery	5	8.1
Radiation	4	6.5
Transplant (including bone marrow and/or stem cells)	2	3.2

Symptom and treatment codes could co-occur, so percentages may not add to 100%

^a^Other included symptoms such as seizure, *n* = 3 (2.8%); slurred or affected speech, *n* = 3 (2.8%); and tremors, *n* = 2 (1.9%)

^b^These symptoms were often shown together during our practice coding, so they were combined

^c^Pills could include oral chemotherapies; the type of oral medication (e.g., oral chemo vs. others) was almost never specified

More than half of these medical scenes did not show the character with cancer receiving any cancer treatment (*n* = 139, 69.2% of medical scenes), and nearly one-half of the medical scenes did not depict cancer or treatment-related symptoms or side effects (*n* = 93, 46.0% of medical scenes). Among scenes that did depict cancer treatment and/or related symptoms, chemotherapy was the most depicted treatment (*n* = 27, 43.5% of scenes with a treatment) and pain was the most depicted symptom/side effect (*n* = 36, 33.3% of scenes with symptoms or side effect).

### Social scenes

There were 374 unique social scenes within the sample. Each movie averaged 19 social scenes, and the amount of screen time devoted to scenes depicting social challenges of cancer ranged from 30.8 to 96.2 min (*M* = 55.1, *SD* = 15.6, median = 51.1). Most movies depicted one or two unique social challenges per scene (*M* = 1.7, *SD* = 0.3).

The *death salience*, *caring*, and *interpersonal interactions* categories were the most frequently used (see Table 4). Popular codes in the death salience category included preparing for death (*n* = 78, 43.8% of death salience category), bucket list (*n* = 25, 14.0%), and after the death of the character with cancer (*n* = 25, 14.0%). Popular codes in the caring category included caregiving (*n* = 73, 44.0% of caring category) and managing others’ emotions (*n* = 52, 31.3%). Popular codes in the interpersonal interactions category included disclosure of diagnosis (*n* = 59, 45.4% of interpersonal interaction category) and dark humor (*n* = 31, 23.9%).

**Table 4:** Frequencies of codes applied to social scenes (*N* = 374) by code and movie

Social scene code	Gwet’s AC_1_	Number	% of codes per scene	Movie with most instances of code	Number	% of total instances of code^a^
Death salience		178	47.6		19	95.0
Preparing for death	0.87	78	43.8	*Irreplaceable You*	13	16.7
Bucket list	0.95	25	14.0	*Then Came You*	11	44.0
After death of character with cancer	0.99	25	14.0	*I Still Believe* & *Irreplaceable You*	4	16.0
Rushed marriage	0.99	16	9.0	*After Everything*	4	25.0
Afterlife (religion)	0.96	13	7.3	*The Fault in Our Stars*	4	30.8
Regret	0.96	10	5.6	*Hope Springs Eternal*	3	30.0
Suicidal ideation	1.00	7	3.9	*Sequoia*	6	30.8
Death of cancer friends	1.00	4	2.2	*Irreplaceable You*, *Life in a Year*, *The Fault in Our Stars*, & *Until Forever*	1	25.0
Caring		166	44.4		20	100.0
Caregiving	0.86	73	44.0	*Our Friend*	12	16.4
Managing others’ emotions	0.90	52	31.3	*The Fault in Our Stars*	13	25.0
Loss of independence	0.97	23	13.9	*The Fault in Our Stars*	7	30.4
Parenting with cancer	0.99	18	10.8	*Our Friend*	8	44.4
Interpersonal interactions		130	34.8		20	100.0
Disclosure of diagnosis	0.89	59	45.4	*Hooking Up*	11	18.6
Use of dark humor in interaction	0.93	31	23.8	*Clouds*	5	16.1
Othering	0.92	22	16.9	*Me and Earl and the Dying Girl*	6	27.3
Unhelpful social reactions	0.97	14	10.8	*Me and Earl and the Dying Girl*	4	28.6
Unwanted medical advice	0.99	4	3.1	*Irreplaceable You*	2	50.0
Processing		78	20.9		18	90.0
Extreme emotions	0.91	37	47.4	*Hope Springs Eternal*	6	16.2
Religion	0.98	25	32.1	*Until Forever*	13	52.0
Fear of missing out	0.94	16	20.5	*Clouds* & *The Fault in Our Stars*	4	25.0
Physical		41	11.0		17	85.0
Physical changes	0.97	34	82.9	*Hooking Up*	5	14.7
Fertility	1.00	7	17.1	*Her Only Choice* & *New Life*	3	42.9
Survivorship		30	8.0		8	40.0
New normal	0.96	25	83.3	*Hope Springs Eternal*	10	40.0
Scanxiety	0.99	5	16.7	*After Everything* & *Hooking Up*	2	40.0

Social scenes could include more than one code, so percentages may not add to 100%. Gwet’s AC_1_ is a chance-corrected agreement coefficient ranging from 0 to 1, with 1 indicating perfect intercoder agreement. Values above 0.80 are thought to be acceptable [31–33]

^a^For categories with more than one movie representing most instances of code, the percentages listed are per movie

Notably, several codes were predominantly in only one or two movies. For instance, more than half of the religious processing code came from scenes in *Until Forever* (*n* = 13, 52.0% of the religious processing code). Exactly half of the scenes containing unwanted medical advice came from *Irreplaceable You* (*n* = 2, 50.0% of the unwanted medical advice code), and more than a third of scenes containing bucket list and parenting with cancer came from *Then Came You* (*n* = 11, 44.0% of the bucket list code) and *Our Friend* (*n* = 8, 44.4% of the parenting with cancer code), respectively.

## Discussion

Results of this content analysis of 20 recent movies featuring depictions of adolescent or young adult cancer indicated that most movies failed to reflect the reality of the AYACS experience in the USA. In the sample, characters with cancer were primarily White, heterosexual females, and more than half had died by the end of the movie. Medical scenes comprised, on average, only about a quarter of the movies’ run times, with less than one-third of those scenes depicting any type of cancer treatment. More than half of the cancer-related social scenes featured death-related themes.

AYACS experiences with cancer in the movies greatly differ from the real world. The diagnosis or specific type of cancer was not clearly stated in nearly one-third of our sample, a finding aligned with earlier analyses of cancer movies [27, 28]. Cancer treatment is notoriously time-intensive, but the proportion of time allotted to medical scenes within each movie is less than the time real-life AYACS spend dealing with the immediate and downstream effects of their illness [36, 37]. Additionally, the survival rate (i.e., the character was still alive at the end of the movie) across the sample was about 42%. Only 20% of characters were depicted as achieving “true” remission, with the strong implication that the remaining 22% would perish at some point in the not-so-distant future. While this survival rate is slightly higher than in previous analyses of cancer movies [27, 28], it is still much lower than the nearly 90% 5-year survival rate for real-life AYACS [29, 38]. The lack of disease survival likely contributed to the sample’s lack of depictions of survivorship-related challenges, which appeared in less than 5% of our sample’s social scenes. Moreover, despite being a common concern among AYACS, depictions of reproductive concerns, including fertility preservation, were rarely addressed within our sample [39]. In fact, just seven of the 374 unique social scenes (1.9%) addressed reproductive concerns, and these scenes were in just three movies.

Thus, considered together, it is possible that the lack of realism within these AYA cancer-focused movies could negatively affect the nearly two million AYACS living in the USA [29]. While surviving cancer is an important milestone for real-world AYACS, the literature is clear that cancer-related challenges are lifelong; long-term survivors and those living with chronic disease face a host of physical, emotional, social, and financial complications well past their initial diagnosis [29]. Prior research has found that this lack of realism is one reason that cancer survivors themselves avoid these movies [21]. Yet movies can be highly influential, especially to those who have little or no exposure to a particular topic [40]. Movies are memorable because they, by design, evoke strong emotions and create vivid mental images [41]. Cognitively, they are also processed similarly to factual narratives, even when labeled as fiction, meaning that they can be perceived as factual for viewers [42]. Our study was not designed to examine the effects of these unrealistic depictions on societal understanding of cancer; however, our findings do suggest that AYA cancer movies show a very different cancer experience than the one experienced by real-world AYACS. The effect of these movies on adolescent and young adults’ perceptions and understandings of the treatment, outcomes, and survivorship of a cancer diagnosis is a prime area for future social support research.

The movies’ inclusion of themes related to caring and dark humor was noteworthy because these themes align with the extant literature on AYACS social experiences of cancer. The caring category was the second most frequent category among our social scenes, and it included social challenges related to *caregiving*, *managing others’ emotions*, *loss of independence*, and *parenting with cancer*. Cancer is widely regarded as a communal disease that affects not only the individual with cancer, but also that person’s immediate caregiver, entire family, and larger social network [43, 44]. Daily caregiving tasks, as well as shifting social roles such as loss of independence or changes to parental identity, are key components of life for an AYA facing cancer [2]. Also notable was the movies’ inclusion of *dark humor*. Dark humor includes rhetorical devices such as sarcasm and absurdity, and it appeared 31 times in the sample [45]. While that is less than 10% of the sample’s social scenes, dark humor is notable as it is a significant characteristic of AYACS coping [11, 14, 45]. Inclusion of these themes could normalize some of the challenges that arise in these domains, as well as potentially help AYACS cope with or make meaning from their diagnosis [21].

### Limitations

There are some limitations to this work. First, though we tried to identify all movies with an AYA cancer storyline released during the study period, there is no singular source of such titles, and exact search dates and search strings were not recorded. Furthermore, only one study team member screened the potentially relevant titles for inclusion. Thus, it is possible that we missed an eligible movie, and/or an eligible movie was not included in the final sample. Additionally, because filmmakers also left room for some interpretation of symptoms (e.g., a mark on a body part could be bruising, but it could also be changes to skin), we may not have captured the true rate of each symptom. This project also investigates and reports on the accuracy of the cancer depictions within movies produced for entertainment. Accuracy, then, may not be a goal for the people who created the movie. We also did not take the additional step of asking AYACS how they felt about the representations of cancer within the movie, although our team has reported results of such investigations elsewhere [19, 21]. Finally, we did not adopt a specific theoretical framework through which to interpret these findings. Future studies may benefit from applying specific psychosocial or communication theories to further interpret or extend our understanding of depictions of AYA cancer in movies.

## Conclusions

In this quantitative and thematic content analysis of movies addressing adolescent and young adult cancer released between 2013 and 2023, we found that movies mostly do not reflect the reality of AYA cancer in the USA, especially the realities of medical treatment and physical and emotional consequences. The movies realistically depicted some social challenges related to cancer treatment, such as caregiving and using dark humor. Cancer survivors should expect to encounter these inaccuracies when they watch these types of movies. However, survivors may also find that pointing out and explaining these inaccuracies are easy ways to start conversations about their experiences with friends. Future research should determine the effect of these portrayals on both survivors and friends. Future research should also explore whether co-viewing cancer-related movies can help survivors and friends have productive cancer-related conversations.

## Supplementary Information

Below is the link to the electronic supplementary material.

ESM 1 (DOCX 243 KB)

## Data Availability

The data are available on request from the corresponding author.
