# Supplementary material for: How adolescent and young adult cancer is portrayed in movies: reflections for supportive care
Source: Support Care Cancer. 2026 Aug 31;34(9):910. doi: 10.1007/s00520-026-11138-3 (PMC13526720; doi:10.1007/s00520-026-11138-3)
Supplement: Supplementary file 1 [file 520_2026_11138_MOESM1_ESM.docx]

# Depictions of Cancer

The intent of this section is to code the characters with cancer in each of the films, as well as how the disease and its treatment processes are depicted.

| **CANCER CHARACTERISTICS: CHARACTER(S) WITH CANCER** | | |
| --- | --- | --- |
| Code EACH leading or supporting character **with cancer** in the film. Any minor characters do not need to be coded. *Any characters that do not have cancer do not need to be coded.* | | |
| Name of character **with cancer** | Write in first name |  |
| Race | 1 = White  2 = Black  3 = Hispanic/Latino  4 = Asian  5 = Multiple races  6 = Other (specified in movie, write-in)  7 = Unclear (to be discussed) | Pavisic, 2014  Character race will be determined by the character’s outward appearance, unless there is something within the movie that contradicts the character’s outward appearance. For example, if the character appears to be White but is shown as attending a family member’s quinceañera, then that character can be coded as Hispanic/Latino.  How would the *average* viewer perceive their race? |
| Gender | 1 = Male  2 = Female  3 = non-binary or non-gendered (specified in movie, write-in)  4 = Unclear (to be discussed) | Character gender is determined using the information provided in the movie description, on the movie case, and in the movie:   - Gender is coded based on the pronouns used by the character. - If no gender pronouns are used, gender is determined by the character’s name. If the name is gender ambiguous, then use context clues (i.e., visual, textual, etc.) to determine the gender. - If gender still can’t be determined, then code as “unclear”. |
| Sexual orientation | 1 = Heterosexual  2 = Not heterosexual  3 = Unclear | Sexual orientation is determined using the information provided in the movie:   - If character is dating or interested in a character of a different gender identity (i.e., NOT the gender they identify with), then code as heterosexual. - If character is dating or interested in a character of the same gender identity (i.e., the gender they identify with), then code as not heterosexual. - Code as UNCLEAR if there is any question as to the character’s sexual orientation. |
| Diagnosis | Write-in, exactly as given in movie | If the specific diagnosis is not given, write “Cancer, not specified”. |
| Prognosis | 1 = Curable  2 = Ambiguous  3 = Terminal  4 = Odds given (specify) | This code refers to the character’s expected survival, **as given upon introduction of cancer**. This can be communicated to the character by a doctor, discussed among a cancer and non-cancer character, or derived from other contextual clues.  If the film shows a character providing odds (e.g. “50/50”), then select the “odds given” option and write in the odds given **exactly as given** in the movie. Make sure to note which odds are associated with survival vs. death (e.g., “60 survival/ 40 death”). |
| State of the character(s) with cancer at the end of the movie | 1 = Remission / No Evidence of Disease (NED)  2 = No change (cancer is neither worse nor better)  3 = Dead  4 = Unclear |  |

| **MEDICAL SCENES** | | |
| --- | --- | --- |
| Code **EACH** medical scene in the movie.  A medical scene is one in which a character is shown interacting with medical personnel or a medical setting, receiving cancer treatment, and/or experiencing symptoms related to the presence of cancer or cancer treatment | | |
| Scene start time |  | Pause at the beginning of each scene and note the time in the movie. For example:  For this example, you would write in **00:54:24** as the start time. |
| Scene end time |  | Pause at the end of each scene and note the time in the movie. For example:  In this example, you would write in **00:54:53** as the end time.   |
| Scene Setting | 1 = Hospital inpatient  2 = Hospital or clinic outpatient  3 = Home  4 = Hospice  5 = Other (write in) | This is the place where the symptoms, treatment, or tests happen.  **Inpatient refers to the character being hospitalized.** That is, if they are shown as in a hospital bed, in a private (or shared) hospital room, in a hospital gown, staying overnight, etc. those are clues as to inpatient.  **Outpatient refers to the character going to the clinic**. They will be in a medical setting for the treatment, but we should see them wearing normal clothes, in a chair or other non-bed seating, going to and from the hospital at the beginning and end of the treatment, etc. those are clues to outpatient. Some tests are commonly done as outpatient procedures, such as a colonoscopy.  **Hospice refers to a specific facility** where the character has moved. It is not a hospital, and it is not their home. *Home hospice* at the character’s (or character’s support person’s) home **should not be included** here. If they are at their home, code as “home”. |
| Symptoms | **Check all that apply:**  1 = Nausea/vomiting/other gut issues  2 = Fatigue  3 = Extreme emotions/Irritability/ mood swings/ difficulty concentrating  4 = Bleeding, bruising, swelling, or lumps  5 = Pain or trouble moving around  6 = Hallucinations or problems with vision or hearing  7 = Fever or illness that doesn’t go away  8 = Trouble breathing or reduced function of other systems (cardio, pulmonary, etc.)  9 = Hair loss  10 = Changes to skin or nails  11 = Other (write in specifics)  12 = No symptoms | ***If you are unsure, include the symptom in the data set for discussion***  If the character says they are feeling something (i.e., stress, worry, emotions, etc.), include it |
| Treatment type | **Check all that apply:**  1 = Surgery  2 = Chemotherapy  3 = Radiation  4 = Pills  5 = Other (specified in movie, write-in)  6 = Not applicable | Treatment type refers to the type of treatment any of the leading or supporting characters with cancer undergo.  When referring to chemotherapy, only check “chemo” if it’s IV-administered, not pills.  If **multiple characters** are seen getting the **same treatment together** (i.e., all getting chemo in the same infusion room), then only code that as one treatment.  Check all that apply should only be used when a character is receiving more than one type of treatment **in the same scene**. |

# Depictions of Social Scenes Related to Cancer

The intent of this section is to code how the films depict social challenges that might arise due to an adolescent or young adult’s cancer diagnosis.

Social scenes are scenes in which a character or characters face a challenge, **caused by the presence of cancer**, in interacting with other characters or social institutions.

| **SOCIAL CHALLENGES** | | | |
| --- | --- | --- | --- |
| Briefly describe the social scene as depicted in the movie. Include which characters are present | Open-ended | See Appendix A for *examples* of cancer-related social challenges (***Not an exhaustive list***). |  |
| Social scene start time | Write-in | Pause at the beginning of each challenge and note the time in the movie. For example:    For this example, you would write in 00:54:24 as the start time.  Start either at the beginning of the challenge (hinting at a challenge fleshed out later in the scene is the beginning of the challenge – like the foreshadowing pain) OR at the beginning of the scene if the challenge is the first thing that happens in the scene. |  |
| Social scene end time | Write in | Pause at the end of each challenge and note the time in the movie. For example:    In this example, you would write in 00:54:53 as the end time. |  |

| **CATEGORIZATION OF SOCIAL SCENES** | |
| --- | --- |
| **Physical Concerns:** This category refers to any challenges that arise from the physical (bodily) ramifications of a cancer diagnosis and its treatment. It does not refer to concerns about death. | |
| Physical Changes | The character with cancer is concerned about the way their body looks or is due to treatment. This would include hair loss. |
| Fertility Concerns | The character with cancer is worried that they will not be able to have children naturally in the future due to their cancer diagnosis and treatment. This would include preparing for that possibility, such as fertility preservation procedures. |
| **Processing:** This category refers to any challenges that arise from the character’s internal processing of the cancer diagnosis and its treatment, and how those challenges affect their behavior in the social world. | |
| Extreme emotions and behavior | The character with cancer displays a recklessness and extreme disregard for physical safety and/or acts on or displays extreme emotions that other characters or a reasonable individual would not feel in the situation. |
| “Fear of Missing Out” (FOMO) | The character with cancer feels "left out" of normal life |
| Religion | The character with cancer relies on their faith to process their situation; this code is applicable even if the character is questioning their religion. *This code is also applicable to* ***other characters*** *relying on their faith or questioning their religion in the face of the cancer.* |
| **Survivorship**: This category refers to any challenges that arise from the character surviving their diagnosis and treatment. | |
| New normal | Character with cancer *and/or other characters* are adjusting back to normal life after intensive treatment |
| Scanxiety | The character with cancer is worried that the cancer has returned before a regular scan or check-up |
| **Death Salience**: This category refers to any challenges that arise from the character with cancer facing their own mortality. | |
| Preparing for death, final days, and death | Character with cancer plans their own funeral and/or engages with other characters in discussions about their funeral. Character with cancer prepares others for their eventual death. The character with cancer is shown as actively dying and/or living their final days. *For example, the character with cancer could also be shown saying goodbye to others. Alternatively, the other characters could arrange a special outing for the character with cancer, etc.* |
| Death of cancer friends | The character(s) with cancer experience the death of a friend, significant other, or other important person who also had cancer. |
| Bucket list | Character with cancer engages in items that they've put on a "bucket list" to experience before they die. *This includes other characters carrying out “bucket list” items on behalf of the character with cancer after their death.* |
| (Rushed) marriage | The character with cancer gets married because of or during cancer treatment. |
| Religion and/or afterlife | Religion may also come up related to death and the afterlife. If the scene mentions or implies death, use the “death salience” code instead of the “processing” code. |
| Suicidal ideation | Contemplating or acting on “beating cancer to the punch” by committing suicide. |
| Regret | *Any character* regrets wasting time that they could have spent with the character with cancer, or the character with cancer regrets something they did not do. |
| After death of character with cancer | The characters who didn’t have cancer are depicted as moving on, dealing with challenges, and/or coping after the death of the character with cancer. |
| **Caring:** This category refers to any challenges that arise from a character caring for another character with cancer, from the character with cancer parenting and/or worrying about their own children, and/or from the character with cancer being cared for. | |
| Parenting with cancer | Relevant to characters who are parents in scenes in which they are depicted as planning for their children's lives without them |
| Caregiving | Social network member engages in concrete caregiving tasks for the character with cancer, such as preparing food, helping with meds, etc. Includes worrying that the care provided is “wrong” or “inadequate”. This could also include social network member providing emotional support, companionship, and/or doing nice things for the character with cancer. This could also include the character with cancer providing concrete caregiving tasks (but NOT emotional support; see other’s emotions) to others. |
| Loss of independence | Character with cancer experiences "overparenting", or what they perceive as too much involvement from their parents due to the diagnosis. |
| Managing others’ emotions | The character with cancer is forced to manage the emotions of others regarding their cancer diagnosis and/or prognosis. This could include providing comfort, but it could also take other forms. |
| **Interpersonal communication:** This category refers to any challenges that arise from the character’s (usually verbal) interactions with one or more other characters. | |
| Diagnosis disclosure | The character with cancer must disclose their diagnosis to others. This includes when the decision to disclose is taken away by others, including them disclosing on the character’s behalf. |
| Unwanted medical advice | Unsolicited advice from healthy characters regarding foods to eat, substances that "kill" cancer, "If you do this, cancer will go away", and/or "You got cancer because of X"; etc. |
| Unhelpful social reactions | "Someone I know died of cancer" |
| Othering | The character with cancer experiences "othering" by people in their social circle due to the cancer diagnosis. This could include bullying, "ghosting", and/or a lack of displayed understanding about the situation. |
| Dark humor | The character with cancer makes jokes about cancer treatment and/or death. *That includes brushing off the seriousness of the situation and/or telling others that it’s not a big deal.* |

**Appendix A: Examples of Cancer-Related Social Challenges**

Previously good friends “ghosting” person with cancer (i.e., not communicating/not seeing the character who is sick)

Trivialization of the situation or bringing up people who’ve died from cancer, especially those who are older (i.e., grandparents, aunts, uncles, etc.)

Missing social gatherings due to the presence of physical limitations imposed by the illness or its treatment (i.e., immunocompromised, extreme fatigue, etc. Be sure to note whether friends display understanding or non-understanding)

Feeling “left out” due to cancer (FOMO – life is continuing but you can’t participate because of the illness)

Others commenting on or bullying due to illness, treatment, or physical changes due to illness and treatment

Parent taking care of an adult previously self-sufficient (loss of independence)

Deciding whether to disclose the cancer diagnosis, including the disclosure decision being taken away from the character with cancer

Recklessness and extreme disregard for physical safety

Pushing others away, being or becoming a “loner” after the diagnosis

Acting on or displaying extreme emotions that other characters do not understand or feel

“Cancer perks” or receiving special treatment due to illness, even if other characters do not resent the special treatment

Saying final goodbyes to others

The character planning for after their death (i.e., sending gifts to friends or family, leaving notes, planning their own funeral, etc.)

Humor (especially dark humor) related to cancer, death, side effects, medical system, etc.

Unsolicited medical advice (including “natural remedies”)

Being the youngest person in treatment

Experiencing the death of cancer friends

Friends or family thinking they should be doing “more” and/or asking for suggestions
